# Acute oxygen desaturation characterizes pulmonary aspiration in patients with gastroesophageal reflux disease and laryngopharyngeal reflux

**DOI:** 10.14814/phy2.15367

**Published:** 2022-06-27

**Authors:** Daminda P. Weerasinghe, Leticia Burton, Peter Chicco, Mark Pearson, Douglas J. Mackey, Gregory L. Falk

**Affiliations:** ^1^ CNI Molecular Imaging & University of Notre Dame Sydney Australia; ^2^ Clinical Technology Service Royal North Shore Hospital Sydney Australia; ^3^ Sydney Heartburn Clinic, Concord Hospital University of Sydney Sydney Australia

**Keywords:** gastro‐esophageal reflux disease, laryngopharyngeal reflux, pulmonary aspiration, pulse oximetry, scintigraphy

## Abstract

The aim of this study was to characterise pulmonary aspiration of refluxate in patients with gastroesophageal reflux disease (GORD) and laryngopharyngeal reflux (LPR) by continuous pulse oximetry (SpO2) during the supine phase of a scintigraphic reflux study. Variables assessed for significance included age, hiatus hernia, frequency, amplitude of reflux and clearance of reflux from the oesophagus/pharynx. The patients included in this study had established GORD and LPR by clinical history. All patients underwent fused three‐ dimensional scintigraphic/ X‐ray computed tomography (CT) and simultaneous continuous pulse oximetry when supine for 30 minutes. A total of 265 patients (40.4% M, 59.6% F) were studied. Mean age of aspirators was 57.0 years and non‐aspirators was 53.5 years. Seven patients had baseline oxygen saturation <95%, with 6/7 showing aspiration by scintigraphy. The remainder had mean baseline saturation of 97.7%. Continuous SpO2 monitoring showed a significant fall in pulmonary aspirators after 20 min of supine acquisition with significant variability. Analysis revealed a cyclic event every 1.5 min in aspirators only. Panel regression analysis showed a significant effect of age, hiatus hernia, pulse rate and reflux frequency on the fall in SpO2. Pulmonary aspiration in patients with LPR and GORD is characterised by acute oxygen desaturation. Variables affecting oxygen desaturation were age, hiatus hernia, pulse rate and reflux frequency. A cyclic event was observed every 1.5 min in aspirators and may be due to reflex homeostatic mechanism attempting to correct perceived hypoxia.

## Graphical abstract

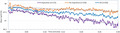

## 1 INTRODUCTION

Pulse oximetry is now routinely utilized for monitoring oxygen saturation (SpO_2_) levels in patients undergoing many surgical procedures under anesthesia. It is also routinely used when monitoring patients with significant systemic illness in the high dependency and intensive care units. It reduces the need for invasive arterial blood gas analysis and provides a fundamental method for continuous monitoring of SpO_2_ with good precision of <3% in readings above 90% (Jubran, 2015). Significant problems have been noted with continuous measurements, in the presence of haemodynamic instability in the intensive care setting (Van de Louw et al., 2001). Hypoxemia in such patients has been defined as an SpO_2_ of <95% (Amoian et al., 2013).

The variability of SpO_2_ measurements in a healthy adult population has been shown to have a standard deviation (*SD*) of 0.71% around a mean of 97.7%, with less variability at higher SpO_2_ levels (Bhogal & Mani, 2017). Intriguingly, the pattern of variability was shown to possess a fractal‐like quality on detrended fluctuation analysis (Peng et al., 1995). No significant difference in mean SpO_2_ or variability was found between the normal young and older age groups enrolled in the study.

We hypothesized that the greater variability in continuous SpO_2_ measurements in patients with gastro‐esophageal reflux disease (GORD) may be due to airway contamination by refluxate. The study group had established GORD with predominantly atypical symptoms suggestive of laryngopharyngeal reflux (LPR) and possible pulmonary aspiration of refluxate. The study prospectively evaluated a novel validated scintigraphic reflux study that has been shown to demonstrate refluxate contaminating the laryngopharynx and lungs (Burton et al., 2018; Burton, Falk, Baumgart, et al., 2020; Burton, Falk, Beattie, et al., 2020; Khoma et al., 2020) with simultaneous continuous supine percutaneous pulse oximetry for 30 min. The aim was to measure the effect of refluxate in the airways on resting oxygen saturation while supine.

## 2 METHODS

### 2.1 Patient group/data

Two hundred and sixty five patients were enrolled in this study for investigation of reflux and associated complications between July 2020 and August 2021.The patients had established GORD/LPR refractory to standard anti‐reflux therapy with predominantly extra‐esophageal symptoms. Predominantly high‐dose proton‐pump inhibitor therapy was empirically prescribed for symptoms with little effect in the vast majority of patients. The patient studied were referred for atypical symptoms of gastro‐esophageal reflux disease and most had been screened historically or by sleep studies before being entered into this study. Patients with obstructive sleep apnoea were enrolled in a separate study. Virtually all patients were tertiary referrals from specialists in respiratory medicine or upper gastrointestinal/ENT surgeons with the principal symptoms of cough and throat discomfort.

Patients with typical GORD symptoms of heartburn and regurgitation only were excluded from the study. The main clinical symptoms were chronic cough, recurrent throat discomfort, globus, recurrent chest infections and intermittent dyspnoea. All patients completed standardized reflux symptom profile questionnaires including the Reflux symptom index (Belafsky et al., 2002), cough severity index (CSI) (Shembel et al., 2013) and Newcastle laryngeal hypersensitivity questionnaire (Vertigan et al., 2014). The symptom of heartburn was reported in <10% of patients. The diagnostic indication of the test was LPR contamination with or without lung aspiration of refluxate. Percutaneous pulse oximetry was simultaneously obtained during the supine phase of the scintigraphic acquisition over a period of 30 min.

### 2.2 Scintigraphy

Patients were fasted for 4 h prior to the study. A dose of 60–100 Mbq of Technetium‐99 m Fyton was administered in 50 ml of water followed by a further 50 ml of water to clear residual radioactivity in the oropharynx and esophagus.

**FIGURE 1:**
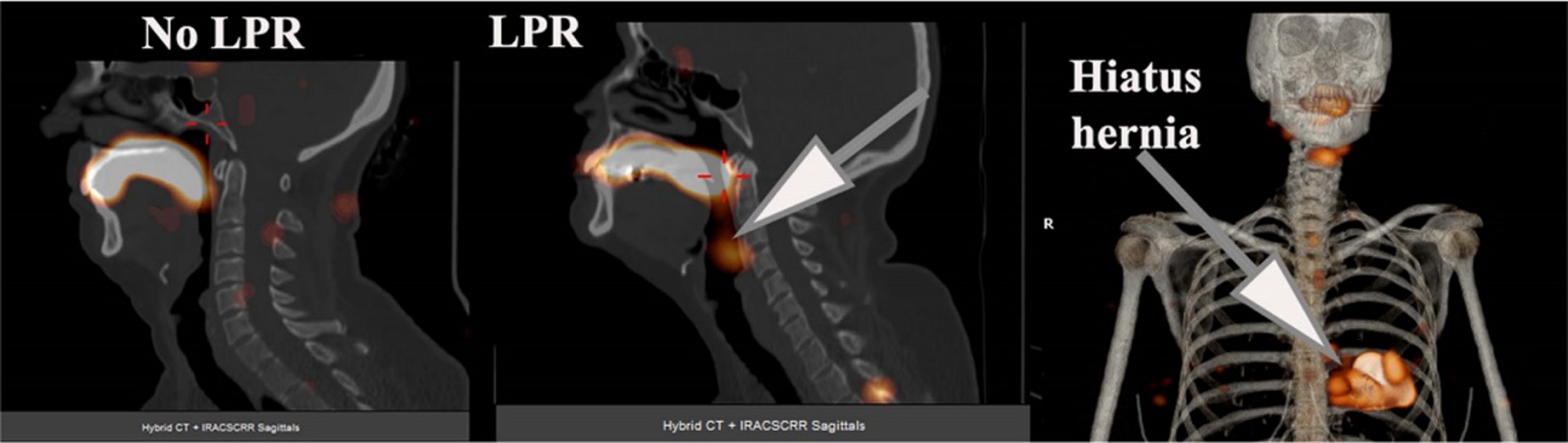
SPECT/CT of sagittal fused images showing a patient with no LPR and the typical pattern of LPR (arrow). The third images is the MIP of the thorax showing a hiatus hernia. LPR, laryngopharyngeal reflux; MIP, maximum intensity projection; SPECT/CT, single‐photon emission computed tomography/computed tomography.

**FIGURE 2:**
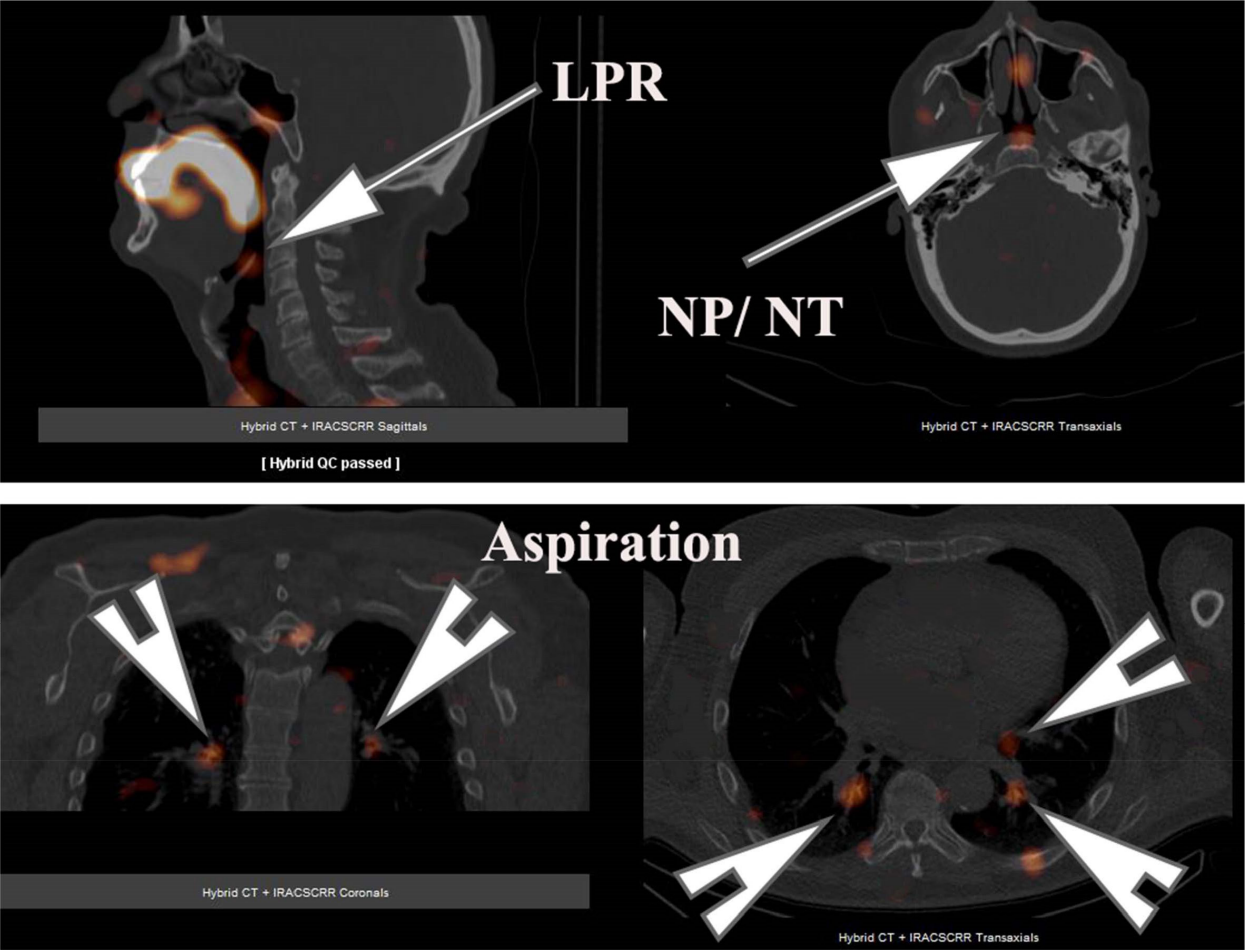
Image of a typical patient with pulmonary aspiration. The upper panel shows fused images with LPR and NP/NT contamination by refluxate (arrows) and aspiration of refluxate into both main bronchi and lingula bronchus (arrowheads). LPR, laryngopharyngeal reflux; NP/NT, nasopharyngeal and nasal turbinate.

Upright dynamic images were obtained at 15 s intervals for 2 min, followed by supine acquisition for 30 min at a similar framing rate. A delayed single‐photon emission computed tomography (SPECT) image of the head, neck and lungs was obtained at 2 h and fused with x‐ray computed tomography (CT) of the region (Figures 1 and 2).

### 2.3 Pulse oximetry

**FIGURE 3:**
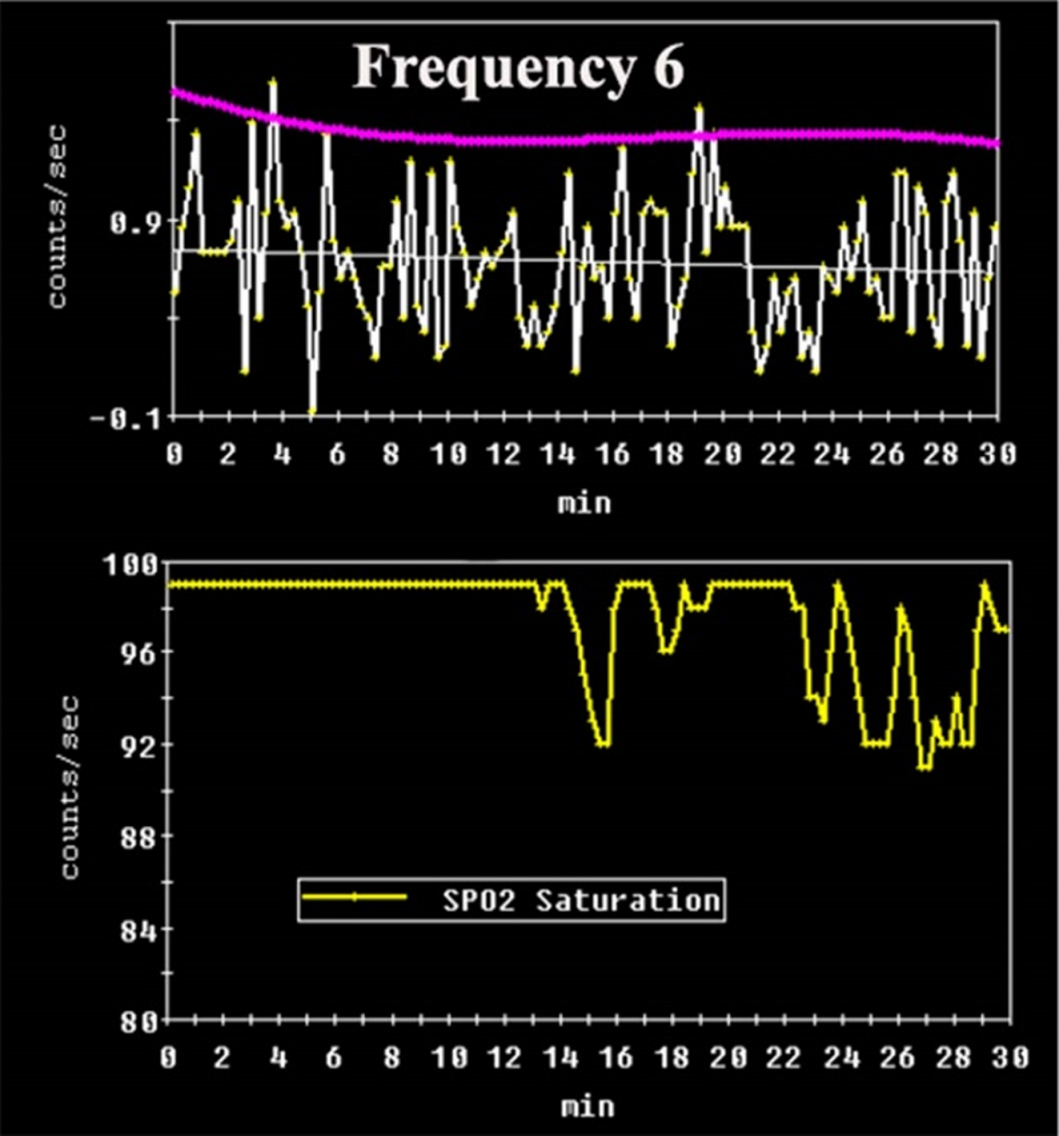
Time‐activity curve from pharynx/laryngopharynx showing episodic reflux above the threshold purple line and the associated decline in oxygen saturation while supine in the lower panel.

A calibrated percutaneous pulse oximeter electrode was attached to either the thumb or middle finger and connected with the acquisition module, which was strapped to the wrist (Wellue SleepU, Viatom Technology). Oxygen saturation measurements were obtained at 4‐s intervals for 30 min and temporally fused with the scintigraphic data (Figure 3). This provided 451 records per patient) and each completed study was stored in a password‐protected Excel spreadsheet.

### 2.4 Statistical analysis

Scintigraphic data assessed included amplitude, area under the curve (AUC) for pharynx to background, frequency of pharyngeal supine reflux (Frequency) and gastric emptying time. Other parameters recorded include patient age, gender, evidence of pulmonary aspiration (aspiration) and hiatus hernia (HH).

Age, amplitude, AUC, GE, Frequency, SpO_2_ and pulse rate were assessed as continuous variables while gender (male as 1), aspiration and HH were set as dichotomous and coded as 1 or 0. Continuous variables were presented as mean and *SD*. Student's *t*‐test was used to determine the relationships between continuous variables and aspiration, assuming the two samples were independent and normally distributed. Changes in discrete variables were analyzed with *ꭓ* ^2^ tests.

Averaging all patients' SpO_2_ and pulse data, trends were assessed between gender, as well as patients with and without aspiration. By averaging patients SpO_2_ and pulse data trends could be analyzed between gender and those with and without aspiration. The continuous sampling of SpO_2_ and pulse rate over time on the same individual, facilitated observations at multiple time points and therefore, panel data regression could be applied. The regression model identified eight variables‐ pulse rate, age, gender, aspiration, HH, amplitude, AUC and Frequency were assessed on the dependent variable SpO_2_.

Hausman specification test was used for testing the consistency of the estimated parameters (Jaba et al., 2013; Torres‐Reyna, 2007).

Converting time‐domain SpO_2_ data to frequency‐domain allowed periodic patterns to be assessed with Fast Fourier Transformation (FFT) (Spiegal, 1961). The SpO_2_ data collected at 4‐s intervals for 30 min were converted to every 1 s for 30 min (ie: 1801 records per patient), thus the cyclic events were assessed with periodogram and spectral density estimates (Kay & Marple, 1981). The periodogram is a volatile and inconsistent estimator of the spectrum, and the spectral density estimate is produced by smoothing the periodogram ordinates with a weighted moving average (SAS Institute Inc, 1999). The SpO_2_ data were assessed for white noise with Fisher's Kappa and Bartlett's Kolmogorov–Smirnov statistics.

### 2.5 Statistical consideration

Statistical analyses were conducted with SAS software version 9.4 (SAS Institute).

### 2.6 Ethical considerations

The study was approved by the institutional ethics committee of the University of Notre Dame (Reference number 015149S). All patients provided informed written consent for the study.

## 3 RESULTS

### 3.1 Patient data

This study comprised 265 patients with established severe GORD and complicating symptoms of LPR. HHs had been diagnosed in 17% prior to referral. This cohort was 107 (40.4%) males and 158 females (59.6%) with a mean age of 57.0 years (aspirators) and 53.5 years (non‐aspirators) and range of 15.8–87.8 years.

Baseline lung disease was shown in 15 patients of this cohort who had abnormal high‐resolution CT scans showing bronchiectasis, air‐trapping, ground‐glass changes or fibrosis. Of these 14/15 had evidence of pulmonary micro‐aspiration in the scintigraphic studies. This is being reported in another study of the relationship between pulmonary disease and micro‐aspiration of refluxate.

SPECT/CT demonstrated pulmonary aspiration in 115 (43.4%).

Seven patients (2.6%) had a resting or baseline SpO_2_ <95% and of these, 6 had scan‐evidence of pulmonary aspiration. The mean pulse rate was 76.5 (*SD* 13.7) beats/min and the mean SpO_2_ was 97.7% (*SD* 1.7) (Table 1). Over the 30‐min study interval, the mean difference in SpO_2_ variation between the baseline and trough values was significantly higher (*p* = 0.001) for aspirators (mean 3.8, *SD* 2.7) compared to non‐aspirators (mean 2.7, *SD* 2.0). Of the 265 patients, 45 (17.0%) had a HH and there was a statistically significant association with aspiration. The scintigraphic variable Frequency of pharyngeal reflux demonstrated a statistically significant association with aspiration. The CSI was significantly higher for aspirating patients (15.6) compared to non‐aspirating patients (11.1). This data is shown in Table 1.

**TABLE 1:** Bivariate association of patient characteristics for patients with and without pulmonary aspiration

Patient characteristics	Aspiration (*n* = 115)		No aspiration (*n* = 150)		*p* value
Categorical variables	*n*	%	*n*	%	
Gender: male	50	43.5	57	38.0	0.368
Hiatus hernia	27	23.5	18	12.0	***0.014***

Continuous variables	Mean	*SD*	Mean	*SD*	*p* value
SpO_2_	97.6	2.0	97.9	1.4	0.166
Pulse rate	77.9	16.0	75.4	11.5	0.270
Age	56.7	16.8	53.5	16.0	0.144
Amplitude	4.1	11.9	2.6	3.4	0.131
AUC	1.6	2.3	1.2	1.1	0.131
Frequency of reflux	3.9	3.4	3.2	0.9	***0.020***
GE	84.7	148.0	90.8	159.4	0.585
RSI index	22.8	9.6	21.0	9.7	0.166
CSI index	15.6	13.1	11.1	11.1	***0.005***
LHQ index	13.7	3.8	13.4	3.8	0.524

Abbreviations: AUC, area under the curve; CSI index, cough severity index; GE, gastric emptying time (minutes); LHQ index, Newcastle laryngeal hypersensitivity questionnaire; RSI index, Belafsky reflux symptom index; *SD*, standard deviation.

The comparison of mean SpO_2_ and mean pulse rate at 5‐min intervals between patients with and without aspiration are presented in Table 2. At the beginning of the assessments, the SpO_2_ readings for both patient groups were similar. The initial sampling demonstrates similar SpO_2_ readings between the two groups. During the 30 min duration a decline in SpO_2_ and increase in pulse rates was observed. A significant difference in SpO_2_ readings between patients with and without aspiration occurred from the 20th minute onward. A marginally higher pulse rate was recorded throughout the sampling period for patients with aspiration in comparison to patients without aspiration. This difference did not reach statistical significance. Table 2 summarizes these findings.

**TABLE 2:** Comparison of mean oxygen saturation (SpO_2_) and mean pulse rate at every 5 min between patients with and without pulmonary aspiration

Reading at	*n* = 265	SpO_2_	Pulse rate
		Mean	Mean
5th minute	Aspiration	97.7	67.5
	No aspiration	97.7	66.9
	*t* value	0.01	0.5
	Pr > |*t*|	0.994	0.619
10th minute	Aspiration	97.4	67.1
	No aspiration	97.5	66.5
	*t* value	0.27	0.5
	Pr > |*t*|	0.784	0.617
15th minute	Aspiration	97.4	66.9
	No aspiration	97.6	65.6
	*t* value	0.84	1.13
	Pr > |*t*|	0.404	0.260
20th minute	Aspiration	97.0	66.1
	No aspiration	97.6	65.2
	*t* value	−1.96	0.73
	Pr > |*t*|	***0.050***	0.464
25th minute	Aspiration	96.8	65.6
	No aspiration	97.5	64.7
	*t* value	−2.69	0.77
	Pr > |*t*|	***0.008***	0.444
30th minute	Aspiration	96.7	65.5
	No aspiration	97.3	64.8
	*t* value	−1.97	0.61
	Pr > |*t*|	***0.049***	0.545

The panel data regression results of SpO_2_ variation at 4 s intervals with selected independent factors are presented in Table 3. The *R* ^2^ value indicates that 3.0% of the SpO_2_ variation is explained by the random effect model and the influence of selected variables. The Hausman test confirms that the estimated model shows random effects.

**TABLE 3:** Panel data regression results of SpO_2_ variation over 30 min, at 4 s intervals, by patients assessed with selected independent predictors

Model description estimation method: fuller and battese variance components (RanTwo)				
Number of cross sections: 265 (patients), time series length: 451 (4 s intervals for 30 min)				
Fit statistics	SSE	94,887.7	DFE	119,505
	MSE	0.794	Root MSE	0.891
	*R*‐square	0.030		
Variance component estimates:	for cross sections	2.172	for error	0.794
Hausman test for random effects	Coefficients	DF	*m* value	Pr > *m*
	1	1	4.08	0.043

Variable	Estimate	StdError	*t* value	Pr > |*t*|
Intercept	109.1	0.409	266.9	<0.0001
Pulse rate	−0.002	0.001	−2.73	***0.006***
Age	−0.015	0.006	−2.62	***0.009***
Aspiration	−0.192	0.189	−1.97	***0.049***
Gender	−0.293	0.195	−1.50	0.133
HH	−0.431	0.254	−2.08	***0.038***
Amp	−0.007	0.022	−0.31	0.758
AUC	0.032	0.104	0.30	0.762
Frequency	−0.135	0.040	−3.37	***0.001***

Abbreviations: Amp, amplitude; AUC, area under the curve; DFE, degrees of freedom for the error, the numbers of the observations in the data set minus the numbers of the parameters; Frequency, frequency of reflux; Hausman test for random effects: H0: the model shows random effects, H1: the model does not show random effects; HH, Hiatus hernia; MSE, mean sum of squares due to errors, SSE, sum of squares due to errors.

Age, aspiration, HH, pulse rate and reflux frequency variables showed a significant negative effect on SpO_2_ between patients over the period of assessment. The greatest effect was from HH with 43.1%, demonstrating a decline in SpO_2_ during the monitoring period. In comparison to patients with no pulmonary aspiration, a 19.2% decline in SpO_2_ was demonstrated in patients with pulmonary aspiration. With a unit increase in age, patients' SpO_2_ level declined by 1.5%. A unit increase in the frequency of reflux at the level of the pharynx was associated with a 13.5% decline in SpO_2_ level. The negative effect between pulse rate and SpO_2_ was marginal (0.2%).

**FIGURE 4:**
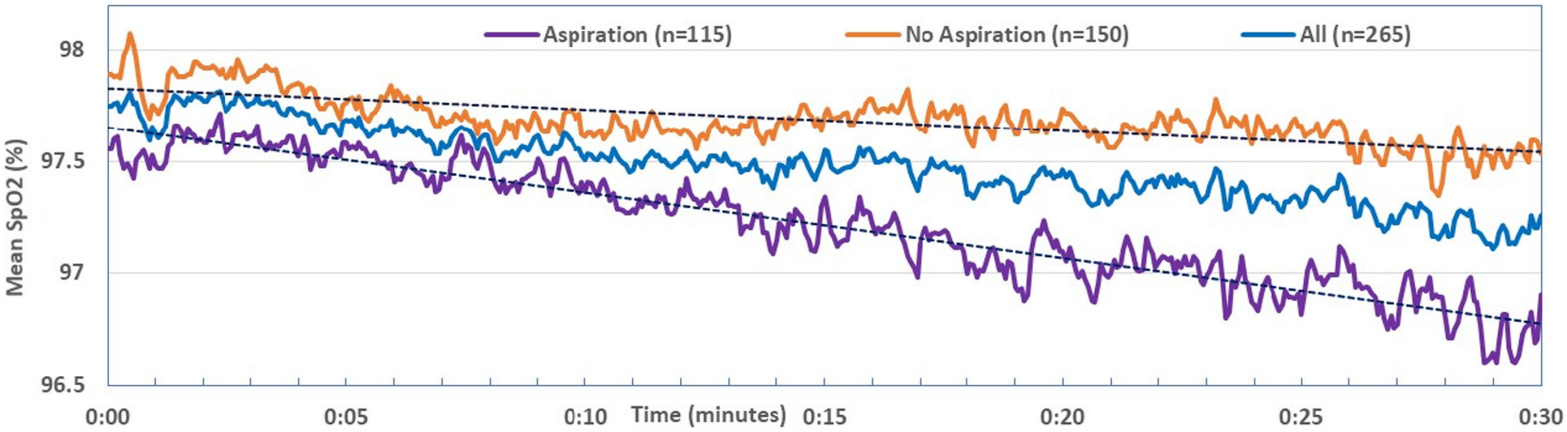
Distribution of mean SpO_2_ during the 30‐min assessment for patients with and without pulmonary aspiration.

**FIGURE 5:**
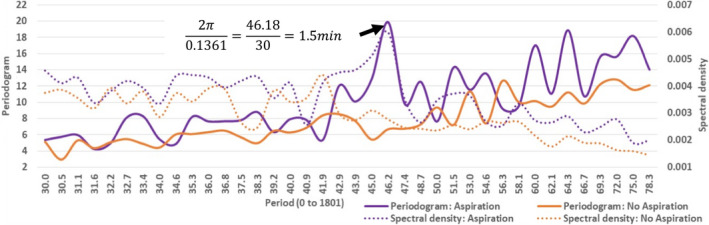
Plots of periodogram and spectral density for SpO_2_ by period for patients with and without pulmonary aspiration (truncated at high frequency domain).

During the 30 min monitoring time, the decline in oxygen saturation was more pronounced in patients with pulmonary aspiration (Figure 4). The time‐series SpO_2_ periodicity was assessed with the plots of the periodogram and spectral density against the period (Figure 5). The SpO_2_ periodicity for patients with pulmonary aspiration showed a distinct peak at the period of 46.2, indicating a SpO_2_ cyclic event around every 1.5 min (92 s). However, no SpO_2_ periodicity was apparent for non‐aspirating patients. The Fisher's Kappa and Bartlett's Kolmogorov–Smirnov tests showed that the series is not white noise (Table 4).

**TABLE 4:** White noise test for SpO_2_ distribution, for patients with and without pulmonary aspiration

	Fisher's Kappa statistic	Bartlett's Kolmogorov–Smirnov Statistic	Approximate *p*‐value
Aspiration (*n* = 115)	4017.0	0.9108	<0.0001
No aspiration (*n* = 150)	7327.1	0.9011	<0.0001
All patients (*n* = 265)	5608.7	0.9067	<0.0001

## 4 DISCUSSION

Blood gas analysis provides important information on oxygenation, carbon dioxide homeostasis and acid–base balance and is a crucial tool for monitoring pulmonary function. However, the technique is invasive, time‐consuming and has a significant lag in analysis. It has largely been supplanted for continuous real‐time monitoring of oxygen saturation by pulse oximetry (Jensen et al., 1998). The technique of utilizing light absorption properties of hemoglobin was first proposed in the 1930s and implemented in 1936 (Severinghaus & Astrup, 1986) with progressive refinements between 1940 and the early 1970s. Further improvements included detection of pulsatile signals and significant advances with microprocessor‐based technologies. The combination of plethysmography and spectrophotometry and the introduction of light emitting diodes in a single probe has revolutionized the technique, with minor variations. Pulse oximetry has subsequently been reported to be accurate to within 5 ± 2% compared to standard in‐vitro arterial blood gas analysis (Kissinger et al., 1991). It has been shown to be most accurate in the 70%–100% range, where readings vary by 1%–2%, compared to standard blood gas evaluation (Hess, 1990). Calibration and maintenance of the devices is critical for the accuracy of the technique.

Only 7 of 265 patients (2.6%) had a resting or baseline SpO_2_ below 95% and of these, 6 had scan‐evidence of pulmonary aspiration, reflecting chronic injury to the parenchymal tissues. All 7 patients had abnormal high‐resolution computed tomography scans. The remainder had a mean baseline SpO_2_ of 97.7% (*SD* 1.7), indicating normal resting oxygenation at the time and accurate measurement by the oximeter. When the data is considered as the greatest deviation from the resting SpO_2_, it shows no significant difference between those with LPR by SPECT/CT only and patients with LPR/pulmonary aspiration (Table 1). The key to the divergence in SpO_2_ between the two groups is the dynamic analysis on a second‐by‐second basis. Divergence is then apparent between aspirators and non‐aspirators (Figure 4) and significant difference in saturation readings occurs after 20 min in the supine position (Table 2). This presumably indicates a lag phase between micro‐aspirated refluxate reaching the main bronchi and pulmonary parenchymal tissues and the resultant effect on gas‐exchange. The commonality of reversible acute oxygen desaturation as a result of micro‐aspiration into the lungs whilst in the supine position in conscious patients is a new and disturbing finding in this study. Patients were unaware of aspiration during the study. It suggests an early temporal relationship between proximal reflux symptoms suggestive of the LPR pattern of GORD and lung disease. This has been suspected in the past but never causally established (Richeldi et al., 2017). The issue of chronic lung fibrosis changing thoracoabdominal pressure differences and inducing gastroesophageal reflux has always been the hypothesized alternative mechanism (Richeldi et al., 2017). The vast majority of these patients present with symptoms long after the genesis of the pathology, evading clear aetiological identification of potential causative elements, such as GORD. Reflux is an important issue as the incidence of “idiopathic” pulmonary fibrosis is rising in North America and Europe (Hutchinson et al., 2015).

There are a number of critical factors that may significantly worsen the effects of GORD on the lungs, contributing to oxygen desaturation. The situation was found to be significantly worse with increasing age, potentially as a result of loss of protective mechanisms in the upper airway and the increasing severity of gastro‐esophageal reflux (Räihä et al., 1992). The presence of HHs was also a significant contributor, which is on the basis that there is disruption of the lower esophageal sphincter mechanism and a large acid pocket within the thorax allows the access of gastric contents into the proximal esophagus, oropharynx and airways in susceptible individuals (Senyk et al., 1975). The increasing frequency of reflux to the pharynx/laryngopharynx was also found to be correlated with pulmonary aspiration and oxygen desaturation. This is the first physical evidence of the importance of the frequency of reflux reaching the upper esophagus and oropharynx and being visualized in the laryngopharynx, increasing the risk of pulmonary aspiration. The literature on esophageal pH/impedance monitoring has implied this finding but never definitively established it as a risk for pulmonary aspiration of refluxate (Oelschlager et al., 2006).

The method of data analysis of the time‐series of oxygen saturation contains a wealth of physiologically important information which has been alluded to in a previous study of oxygen saturation in normal volunteers (Bhogal & Mani, 2017). Panel data regression is a relatively new technique that combines both a cross‐sectional analysis together with the sequential time‐series, in order to demonstrate hidden variables that may be relevant to the dependent variable (SpO_2_) (Jaba et al., 2013; Torres‐Reyna, 2007). Panel data regression allows for control of variables that cannot be observed or measured (Torres‐Reyna, 2007). Cross‐sectional data analysis is fundamentally a method of describing one observation of multiple objects and corresponding variables at a specific point in time. As a safety measure, the result is also tested for the possibility of random occurrence in the estimated parameters (Jaba et al., 2013; Torres‐Reyna, 2007). This analysis found that the variables of age, aspiration, HH, increased pulse rate and pharyngeal reflux frequency show a significant negative effect on SpO_2_ over time. The largest effect on SpO_2_ was from HHs, which were responsible for 43.1% of the effect.

Gas exchange is affected by a multitude of factors, which extend from the molecular content of inspired gas, to the matching level of perfusion of the alveoli for carriage of oxygenated hemoglobin. In‐between are a variety of neuromuscular control and feed‐back mechanisms, that maintain oxygen saturation in a narrow optimal zone (Wagner, 2015). Ultimately, respiratory motor output depends on the integrated properties of the complex central respiratory neural networks to restore tissue oxygenation. Figure 4 demonstrates that as oxygen saturation begins to fall with aspiration of refluxate into the lungs (purple line), there are significantly wider swings in the readings after 20 min, compared to the mean (blue line) and LPR by SPECT/CT only (orange line) graphs. Generally, physiological signals are quite complex due to a variety of influences and behave as non‐linear systems which are best analyzed in the frequency domain rather than the time‐domain (Freeman, 1992; Glass & Mackey, 1988). These changes are best illustrated by converting from the time‐domain to the frequency‐domain by FFT. Figure 5 demonstrates that patients with pulmonary aspiration have a SpO_2_ periodicity of approximately 1.5 min (92 s). This is in contrast to non‐aspirating patients, who demonstrated no SpO_2_ periodicity. This variability may be attributed to reflex homeostatic mechanism attempting to correct oxygen desaturation.

A significant body of physiology literature has noted that the sympathetic nervous system is activated by oxygen desaturation but not hypercapnia (Xie et al., 1985). Activity is rapidly restored to normal by administration of oxygen, suggesting that this may be due to sensitisation of the peripheral chemoreceptors, plus or minus the central nuclei in the chemo‐reflex loops (Querido et al., 1985; Xie et al., 1985). However, if not restored to normal levels by exogenous oxygen (hyperoxia), sympathetic activity remains elevated for a prolonged time after the hypoxic stimulus has recovered. This phenomenon is reflected in the increased pulse rate findings in the sub‐group that aspirated refluxate into the lungs. These effects are modulated by the frequency and amplitude of the sympathetic activation provoked by the degree of oxygen desaturation (Steinback & Kevin Shoemaker, 2012).

## 5 CONCLUSION

In the study sample 97.4% patients had a baseline mean SpO_2_ of 97.7%, indicating normal resting pulmonary function at the time. Only 7 (2.6%) patients had a resting or baseline SpO_2_ below 95% and of these, 6 had scan‐evidence of pulmonary aspiration, reflecting chronic injury to the parenchymal tissues. This study showed a significant divergence in saturation readings in patients with pulmonary aspiration after 20 min when supine. This is a new finding, confirming the commonality of reversible acute oxygen desaturation as a result of micro‐aspiration into the lungs in patients with the severe LPR variant of GORD when supine. It suggests an early temporal relationship between GORD and lung disease. Age, HHs and reflux frequency are key variables that increase the risk of micro‐aspiration into the lungs and measurably alter gas‐exchange in patients with GORD. There is greater variability of SpO_2_ periodicity in patients who aspirate refluxate and this may be due to reflex homeostatic mechanisms attempting restitution of oxygenation with a periodicity of 1.5 min.

## AUTHOR CONTRIBUTIONS

Weerasinghe, Daminda: Writing of manuscript, data analysis and statistical processing. Burton, Leticia: Conception & design of project/writing of manuscript. ASAcquisition and processing of scintigraphic studies. Pearson, Mark Revision of manuscript and data checking. Design of oximetry acquisition and writing of software for acquisition. Mackey, Douglas J: Design of oximetry acquisition and writing of software for acquisition and co‐ordination of scintigraphic study with oximetry study. Falk, Gregory L Conception & design of project/writing of manuscript.

## ETHICS STATEMENT

The study was approved by the institutional ethics committee of the University of Notre Dame (Reference number 015149S) and all the procedures were conducted in conformity with the Declaration of Helsinki. All patients provided informed written consent for the study.

## Funding information

No external funding was received for this study. No corporate involvement was sought or received.

## CONFLICT OF INTEREST

All authors declare that there is no conflict of interest or competing interest in the production of the manuscript.
